# Supplementary material for: MetaRibo-Seq measures translation in microbiomes
Source: Nat Commun. 2020 Jun 29;11:3268. doi: 10.1038/s41467-020-17081-z (PMC7324362; doi:10.1038/s41467-020-17081-z)
Supplement: Supplementary file 10 — Supplementary Data 7 [file 41467_2020_17081_MOESM10_ESM.zip › File2/Confidence_High_Taxonomy/275464_out.krona.html]

Javascript must be enabled to view this page.

members
magnitude
magnitudeUnassigned
count
unassigned
taxon
rank

275464\_out

194

superkingdom
2
194

1224
phylum
1

1
class
28216

1
order
80840

family
995019
1

2026794
species

SRS147614\_contig\_number\_2261
1

phylum
976
193

200643
class
193

193
171549
order

185
2005525
family


SRS012849\_contig\_number\_3813SRS012969\_contig\_number\_contig-100\_2619.240066SRS014683\_contig\_number\_contig-100\_1630.1630SRS017433\_contig\_number\_15251SRS018623\_contig\_number\_contig-100\_6730.104859SRS019808\_contig\_number\_5214SRS023715\_contig\_number\_22658SRS024388\_contig\_number\_9753SRS045004\_contig\_number\_25145SRS045645\_contig\_number\_32682SRS047433\_contig\_number\_10164SRS048164\_contig\_number\_30747SRS049995\_contig\_number\_28906SRS050998\_contig\_number\_3046SRS052697\_contig\_number\_34685SRS054352\_contig\_number\_contig-100\_2266.136892SRS056537\_contig\_number\_26546SRS063489\_contig\_number\_contig-100\_1205.1205SRS075773\_contig\_number\_12513SRS075821\_contig\_number\_4249SRS077392\_contig\_number\_42398SRS078176\_contig\_number\_contig-100\_1140.101758SRS078665\_contig\_number\_10321SRS1041136\_contig\_number\_13012SRS1055069\_contig\_number\_12084SRS142503\_contig\_number\_34475SRS143085\_contig\_number\_2103SRS150029\_contig\_number\_18485SRS893187\_contig\_number\_18641
29
185
genus
375288


SRS1041132\_contig\_number\_6442
658662
species
1


SRS015217\_contig\_number\_23842SRS893382\_contig\_number\_1422
1897013
species
2


SRS011084\_contig\_number\_11349SRS011084\_contig\_number\_21249SRS012273\_contig\_number\_3919SRS013098\_contig\_number\_37143SRS013800\_contig\_number\_25160SRS014235\_contig\_number\_27889SRS014412\_contig\_number\_19613SRS014459\_contig\_number\_18257SRS014948\_contig\_number\_1907SRS014979\_contig\_number\_20757SRS015579\_contig\_number\_23900SRS015663\_contig\_number\_36630SRS015782\_contig\_number\_43650SRS015782\_contig\_number\_contig-100\_1472.223425SRS015794\_contig\_number\_2047SRS015890\_contig\_number\_13763SRS015960\_contig\_number\_4257SRS016095\_contig\_number\_21905SRS016989\_contig\_number\_9559SRS017191\_contig\_number\_14984SRS017701\_contig\_number\_4693SRS018313\_contig\_number\_11293SRS018541\_contig\_number\_5028SRS018836\_contig\_number\_26611SRS018888\_contig\_number\_contig-100\_94.49650SRS018984\_contig\_number\_14510SRS019030\_contig\_number\_13748SRS019178\_contig\_number\_4807SRS019267\_contig\_number\_2759SRS019397\_contig\_number\_3352SRS019601\_contig\_number\_22659SRS019685\_contig\_number\_41984SRS020233\_contig\_number\_33433SRS020328\_contig\_number\_29072SRS021219\_contig\_number\_1167SRS021948\_contig\_number\_16869SRS023526\_contig\_number\_contig-100\_1304.222151SRS023583\_contig\_number\_5779SRS023829\_contig\_number\_490SRS024132\_contig\_number\_24099SRS024435\_contig\_number\_37918SRS043701\_contig\_number\_11784SRS043768\_contig\_number\_18484SRS044535\_contig\_number\_17219SRS045195\_contig\_number\_2147SRS045713\_contig\_number\_11669SRS045826\_contig\_number\_5881SRS046369\_contig\_number\_983SRS047044\_contig\_number\_856SRS049773\_contig\_number\_2515SRS049900\_contig\_number\_contig-100\_287.121305SRS050026\_contig\_number\_12088SRS050422\_contig\_number\_contig-100\_348.181861SRS050422\_contig\_number\_contig-100\_314.192392SRS050752\_contig\_number\_29661SRS051610\_contig\_number\_8640SRS051882\_contig\_number\_7693SRS052027\_contig\_number\_2550SRS052078\_contig\_number\_1020SRS052078\_contig\_number\_3422SRS053214\_contig\_number\_15235SRS053398\_contig\_number\_contig-100\_873.144421SRS053573\_contig\_number\_1335SRS053649\_contig\_number\_11820SRS054590\_contig\_number\_412SRS056259\_contig\_number\_12623SRS057478\_contig\_number\_9255SRS058070\_contig\_number\_19454SRS062701\_contig\_number\_7783SRS063040\_contig\_number\_25406SRS064645\_contig\_number\_236SRS065397\_contig\_number\_2726SRS065504\_contig\_number\_27585SRS074964\_contig\_number\_469SRS075398\_contig\_number\_3109SRS075878\_contig\_number\_6289SRS076804\_contig\_number\_3377SRS076929\_contig\_number\_4523SRS077127\_contig\_number\_2280SRS077454\_contig\_number\_2669SRS077730\_contig\_number\_12548SRS077849\_contig\_number\_31886SRS078419\_contig\_number\_5477SRS097920\_contig\_number\_15748SRS098571\_contig\_number\_43956SRS100021\_contig\_number\_10166SRS103987\_contig\_number\_4507SRS104036\_contig\_number\_6774SRS1041036\_contig\_number\_2810SRS1041039\_contig\_number\_11887SRS1041091\_contig\_number\_19765SRS1041092\_contig\_number\_6717SRS1041095\_contig\_number\_5344SRS1041133\_contig\_number\_19611SRS1041138\_contig\_number\_10300SRS1041142\_contig\_number\_8658SRS1041147\_contig\_number\_1550SRS1055038\_contig\_number\_4578SRS142712\_contig\_number\_2941SRS143148\_contig\_number\_11006SRS143876\_contig\_number\_39952SRS144362\_contig\_number\_27749SRS144506\_contig\_number\_25337SRS144603\_contig\_number\_contig-100\_7547.76650SRS145497\_contig\_number\_20582SRS146764\_contig\_number\_34255SRS146813\_contig\_number\_26378SRS148196\_contig\_number\_28254SRS148784\_contig\_number\_24424SRS148874\_contig\_number\_12051SRS149244\_contig\_number\_9451SRS893253\_contig\_number\_1998SRS893256\_contig\_number\_2914SRS893259\_contig\_number\_4723SRS893285\_contig\_number\_1610SRS893288\_contig\_number\_1245
species
823
116

1
species
1263094

SRS143722\_contig\_number\_810

18

SRS015431\_contig\_number\_12572SRS019068\_contig\_number\_25799SRS019968\_contig\_number\_15639SRS020233\_contig\_number\_22545SRS020622\_contig\_number\_5600SRS045244\_contig\_number\_7521SRS051031\_contig\_number\_6311SRS056519\_contig\_number\_7483SRS058770\_contig\_number\_3784SRS063370\_contig\_number\_8354SRS075878\_contig\_number\_contig-100\_709.150326SRS098717\_contig\_number\_18902SRS1041090\_contig\_number\_7414SRS1041141\_contig\_number\_8315SRS147271\_contig\_number\_39099SRS148721\_contig\_number\_42979SRS148970\_contig\_number\_56SRS893252\_contig\_number\_7196
2293123
species


SRS013158\_contig\_number\_4644SRS013951\_contig\_number\_11370SRS019496\_contig\_number\_13099SRS050925\_contig\_number\_10732SRS1041145\_contig\_number\_contig-100\_308.110012SRS104693\_contig\_number\_9536SRS1055099\_contig\_number\_contig-100\_2553.39720
species
1262912
7

species
2293119

SRS1041137\_contig\_number\_22718
1


SRS014287\_contig\_number\_11479SRS015065\_contig\_number\_39143SRS016056\_contig\_number\_2844SRS019068\_contig\_number\_84735SRS143523\_contig\_number\_23900
species
46503
5


SRS017103\_contig\_number\_16346SRS1041140\_contig\_number\_11016
species
2293114
2

1
387661
species

SRS077127\_contig\_number\_11170

2

SRS022713\_contig\_number\_7752SRS023971\_contig\_number\_881
species
469591

1853231
family
1

574697
genus
1


SRS143342\_contig\_number\_17454
species
1472416
1

1
171550
family

1
genus
239759

1

SRS022071\_contig\_number\_31448
28117
species

815
family
6

816
genus
6

1
457391
species

SRS148511\_contig\_number\_12777

1

SRS064973\_contig\_number\_2214
species
457387

1

SRS893231\_contig\_number\_253
species
820

2

SRS098717\_contig\_number\_12568SRS893342\_contig\_number\_3938
species
817

1
818
species

SRS104311\_contig\_number\_879
